# Navigating Novel Viral Challenges: Understanding, Tracking, and Mitigating Emerging Threats

**DOI:** 10.3390/microorganisms12040807

**Published:** 2024-04-17

**Authors:** Francesco Branda, Massimo Ciccozzi

**Affiliations:** Unit of Medical Statistics and Molecular Epidemiology, Università Campus Bio-Medico di Roma, 00128 Rome, Italy; m.ciccozzi@unicampus.it

The emergence of new viral threats continues to pose significant challenges to global health security. As we have seen with the COVID-19 pandemic, the rapid spread of previously unknown viruses can have devastating consequences, causing widespread morbidity, mortality, and socioeconomic disruption [1]. The COVID-19 pandemic, caused by the novel severe acute respiratory syndrome coronavirus 2 (SARS-CoV-2), has resulted in more than 7 million deaths globally, as of 3 March 2024 [2]. Beyond the direct health impacts, the pandemic has had far-reaching effects on various aspects of society, including health systems, economies, and social structures [3]. For example, the economic fallout of the pandemic has led to significant job losses, business closures, and disruption of global supply chains, exacerbating existing inequalities and pushing millions of people into poverty [4]. In addition, the widespread implementation of lockdowns and social distancing measures caused profound psychological consequences, with increased rates of anxiety, depression, and other mental health problems reported globally [5].

This Special Issue, entitled “Navigating New Viral Challenges: Understanding, Tracking, and Mitigating Emerging Threats”, aims to provide a comprehensive overview of the current state of knowledge on new viral threats, and to highlight the importance of proactive measures that go beyond traditional public health strategies [6] to safeguard collective health security. Emerging viral diseases are often the result of complex interactions between viruses, hosts, and the environment. Understanding the molecular mechanisms underlying viral spillovers and adaptations to new hosts is critical for predicting and preventing future epidemics [7]. Viral spillover events, in which viruses move from animal reservoirs to human populations, are often driven by factors such as habitat destruction, climate change, and increased human–animal interactions [8]. Once a virus has spread, its ability to adapt and transmit effectively between humans is influenced by its genetic makeup and the immune responses of the new host species. Recent advances in genomic sequencing and bioinformatics have revolutionized the ability to characterize new viruses and follow their evolution in real time [9]. Techniques such as next-generation sequencing and phylogenetic analysis have enabled researchers to rapidly identify and track the spread of emerging viral threats, as exemplified by the use of genomic epidemiology during the COVID-19 pandemic [10]. By analyzing the genetic diversity and evolutionary dynamics of emerging viruses, we can gain valuable insights into their origins, routes of transmission, and potential for interspecific transmission. For example, genomic analyses of SARS-CoV-2 revealed that the virus probably originated in bats, and was then passed to humans through an intermediate animal host, probably pangolins [11]. In addition, the identification of specific mutations in the viral genome helped explain the increased transmissibility of some SARS-CoV-2 variants, such as the Alpha and Delta variants [12]. This knowledge has informed public health policies and the development of diagnostic tests, therapies, and vaccines.

Effective surveillance systems are essential for the early detection and monitoring of emerging viral threats. The integration of traditional epidemiological methods with cutting-edge technologies, such as next-generation sequencing and big data analysis, has greatly improved the ability to identify and respond to epidemics [13]. Traditional surveillance methods, such as case reporting and contact tracing, remain critical for tracking the spread of diseases and implementing containment measures. However, the incorporation of modern techniques has enabled more rapid and comprehensive monitoring of viral threats. For example, the use of digital tools for disease detection, which leverage data from online sources such as social media, news, and search engine queries, has enabled the early identification of potential outbreaks and the monitoring of public sentiment and behavior [14]. In addition, wastewater monitoring has proven to be a valuable surveillance tool, as it can detect the presence of viral pathogens in wastewater samples before clinical cases are reported, providing an early warning system for outbreaks [15]. The application of advanced analytical and machine learning techniques has also shown promise for improving outbreak predictions and risk assessments [16]. By integrating diverse data sources, such as epidemiological data, genomic data, environmental factors, and human mobility patterns, machine learning models can identify patterns and trends, enabling more accurate predictions of disease spread and the identification of high-risk areas for targeted interventions.

Mitigating the impact of emerging viral threats requires a multifaceted approach, which includes prevention, control, and treatment strategies. Vaccine development is a critical component of pandemic preparedness, and this Special Issue aimed to receive articles on the latest advances in vaccine design, production, and distribution [17]. Developing vaccines that are safe and effective against new viral threats is a complex and lengthy process, involving several stages of research, clinical trials, and regulatory approvals. Major challenges in vaccine development are the rapid identification of appropriate antigenic targets and the design of vaccine candidates capable of eliciting a robust and sustained immune response. Advances in structural biology, computational modeling, and immunology have facilitated the development of innovative vaccine platforms, such as mRNA vaccines and viral vector-based vaccines, which have shown promise in addressing emerging viral threats. In addition, efforts are underway to develop universal or broadly protective vaccines that could provide immunity against multiple strains or even several viral families, reducing the need for strain-specific vaccines [18]. Such vaccines would be valuable in responding to future pandemics caused by previously unknown viruses. Ensuring equitable access to vaccines is another critical challenge, as highlighted by the disparities in COVID-19 vaccine distribution, particularly in low- and middle-income countries [19]. Strategies to address these inequalities include enhancing global production capacities, strengthening supply chains, and promoting international cooperation and resource sharing. Furthermore, addressing vaccine hesitancy and building public trust in vaccination programs is crucial for achieving high coverage rates and reducing the risk of outbreaks [20]. Effective communication strategies, community engagement, and addressing misinformation and disinformation are essential components of successful vaccination campaigns.

In addition to vaccines, the development of broad-spectrum antivirals and host-directed therapies holds promise for the treatment of infections caused by novel viruses [21]. Broad-spectrum antivirals target conserved viral or host factors shared by multiple virus families, potentially offering a more versatile therapeutic option than virus-specific therapies. Host-directed therapies, on the other hand, aim to modulate the host immune response or cellular pathways involved in viral replication, offering an alternative approach to combat emerging viral threats. Research on the identification of potential drug targets, repurposing of existing drugs, and the use of monoclonal antibodies will be highlighted in this collection. The COVID-19 pandemic has accelerated efforts in this area, with several repurposed drugs and monoclonal antibodies showing efficacy in treating SARS-CoV-2 [22,23] infections.

An effective response to epidemics relies on strong interdisciplinary collaborations and the integration of knowledge from diverse fields, including virology, epidemiology, ecology, and the social sciences [24]. Addressing emerging viral threats requires a holistic understanding of the complex interplay of biological, environmental, and social factors that contribute to the emergence and spread of these pathogens. A One Health approach, which recognizes the interconnectedness of human, animal, and environmental health, is essential for addressing emerging viral threats [25]. This approach involves collaboration among experts from various disciplines, including human and veterinary medicine, ecology, wildlife biology, and environmental science, to develop comprehensive strategies for disease prevention, surveillance, and control. For example, investigation of zoonotic viral outbreaks often requires a multidisciplinary team to study the virus, identify animal reservoirs, understand the causes of spillover events, and develop interventions to interrupt transmission cycles [26]. Ecological studies can shed light on environmental factors, such as changes in land use and climate patterns, that may influence the occurrence and spread of viral diseases. In addition, social scientists play a crucial role in understanding human behavior, the cultural practices, and the socioeconomic factors that contribute to the disease transmission and the acceptance of public health interventions. Effective risk communication and community engagement strategies based on social science research are essential for building public trust and ensuring compliance with the control measures.

Case studies of successful collaborative efforts in outbreak investigation and response will provide valuable lessons for future preparedness efforts. For example, the response to the Ebola virus outbreak in West Africa (2014–2016) highlighted the importance of interdisciplinary collaborations and the integration of local knowledge and community engagement efforts in controlling the spread of the disease. Furthermore, the role of international cooperation and global partnerships in coordinating responses to emerging viral threats cannot be overstated. The COVID-19 pandemic has underscored the need for a coordinated global response, involving the sharing of data, resources, and expertise across borders.

In summary, the Special Issue “Navigating Novel Viral Challenges: Understanding, Tracking, and Mitigating Emerging Threats” aims to advance scientific understandings and operational capabilities in addressing viral diseases. Through a meticulously curated collection of research articles, critical reviews, and innovative perspectives, key scientific aspects of viral emergence will be explored, including the molecular basis of viral spreads, the dynamics of transmissions, and the complexities of host–pathogen interactions. Furthermore, by integrating cutting-edge research from disciplines such as virology, epidemiology, and bioinformatics, it intends to highlight the critical role of interdisciplinary approaches in enhancing disease surveillance, improving diagnostic methodologies, and developing robust preventive and therapeutic strategies.

In conclusion, this Special Issue aims to inspire continued research and dialogue within the scientific community to address the evolving challenges posed by new viruses. Collective efforts should focus on strengthening global health security through science-based policymaking, innovations in disease monitoring and control, and the promotion of international collaboration.
